# Stability of Discontinuous Galerkin Spectral Element Schemes for Wave Propagation when the Coefficient Matrices have Jumps

**DOI:** 10.1007/s10915-021-01516-w

**Published:** 2021-05-20

**Authors:** David A. Kopriva, Gregor J. Gassner, Jan Nordström

**Affiliations:** 1 Department of Mathematics, The Florida State University, Tallahassee, FL 32306 USA; 2 Computational Science Research Center, San Diego State University, San Diego, CA USA; 3 Department for Mathematics and Computer Science, Center for Data and Simulation Science, University of Cologne, Weyertal 86-90, 50931 Cologne, Germany; 4 Department of Mathematics, Applied Mathematics, Linköping University, 581 83 Linköping, Sweden; 5 Department of Mathematics and Applied Mathematics, University of Johannesburg, P.O. Box 524, Auckland Park, 2006 South Africa

**Keywords:** Discontinuous Galerkin spectral element, Stability, Linear advection, Discontinuous coefficients

## Abstract

We use the behavior of the $L_{2}$ norm of the solutions of linear hyperbolic equations with discontinuous coefficient matrices as a surrogate to infer stability of discontinuous Galerkin spectral element methods (DGSEM). Although the $L_{2}$ norm is not bounded in terms of the initial data for homogeneous and dissipative boundary conditions for such systems, the $L_{2}$ norm is easier to work with than a norm that discounts growth due to the discontinuities. We show that the DGSEM with an upwind numerical flux that satisfies the Rankine–Hugoniot (or conservation) condition has the same energy bound as the partial differential equation does in the $L_{2}$ norm, plus an added dissipation that depends on how much the approximate solution fails to satisfy the Rankine–Hugoniot jump.

## Introduction

In wave propagation problems, it is natural to find interfaces where material properties like the wave propagation speeds or density abruptly change. Examples include interfaces between two dielectrics in electromagnetic wave propagation problems, or different rock layers in geophysics. At such interfaces the solutions can make discontinuous jumps, causing difficulties for many numerical methods.

One of the key features of discontinuous Galerkin (DG) methods is that the discontinuous approximation at element interfaces naturally allows jump discontinuities in the solution if element boundaries are placed along them. Consequently, DG spectral element methods have been used for over twenty years to solve problems with material discontinuities, both stationary [3, 6, 9, 19] and moving [20]. Computations and theory in such works show that placing the discontinuities at element boundaries leads to exponentially convergent approximations.

In a paper on discontinuous interface problems, La Cognata and Nordström [13] noted that hyperbolic problems with discontinuous coefficients do not necessarily have their energy bounded in terms of the initial data when measured in the $L_{2}$ norm, even with constant coefficients and homogeneous and dissipative boundary conditions. Instead, the $L_{2}$ norm can increase or decrease, depending on the relative size of the wave speeds on either side of the discontinuity. The lack of a bound on the $L_{2}$ norm is not due to an instability in the usual sense, but is due to the fact that conservation at the interface, and the resulting jump in the solution, can increase the norm of the solution as a wave propagates across it. In an alternate norm, however, one that discounts the effect of the jump, the energy is bounded.

Here we propose a procedure where we use the $L_{2}$ norm as a surrogate to infer stability of discontinuous Galerkin spectral element methods (DGSEM) for the approximation of hyperbolic equations with discontinuous coefficient matrices. The $L_{2}$ norm is easier to work with since it does not require finding the discount factors, which are difficult to compute in general configurations of elements and interfaces. We show that the DGSEM with an upwind numerical flux that satisfies the Rankine–Hugoniot (or conservation) condition behaves as the partial differential equation (PDE) does in the $L_{2}$ norm, plus an added dissipation that depends on how much the approximate solution fails to satisfy the Rankine–Hugoniot jump.

## Linear Hyperbolic Systems with Discontinuous Coefficients

In this paper we establish the stability of a discontinuous Galerkin spectral element approximation to linear hyperbolic systems of equations of the form1where $$\mathbf{u}$$ is the state vector, and  is the vector of fluxes,2with coefficient matrices $$ {A}_{j}$$ and unit coordinate vectors $$\hat{x}_{j}$$. We assume throughout this paper that the coefficient matrices are piecewise constant, with discontinuities marking what we will refer to in this paper as *material interfaces*. To isolate the contribution of the discontinuities, we choose the coefficient matrices constant between material interfaces to avoid the complexity of possible exponential growth in the solution norm when the matrices vary over the domain.

**Fig. 1:**
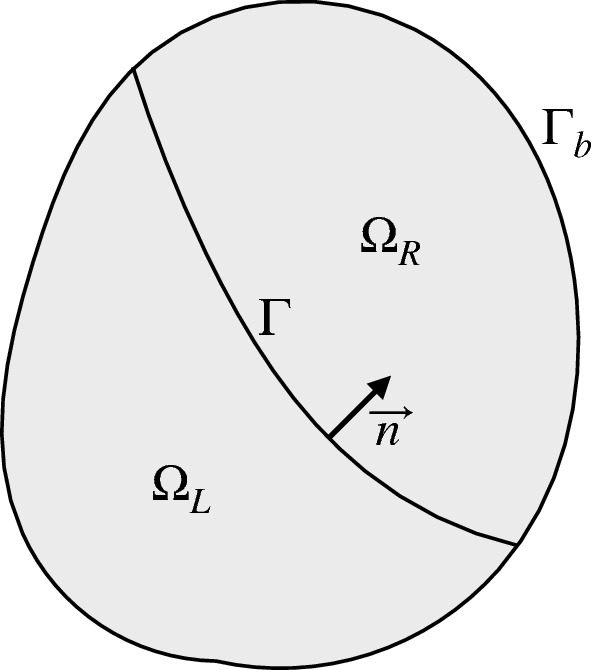
Diagram of a domain ${\varOmega }$ split by a material interface, ${\varGamma }$

We examine the problem defined in a domain ${\varOmega }$, as sketched in two space dimensions in Fig. 1. It is sufficient to consider two domains with a single material interface, so the domain is split into two subdomains ${\varOmega }_{L}$ and ${\varOmega }_{R}$ separated by an interface ${\varGamma }$. The external boundary is ${\varGamma }_{b}$, along which we assume that proper, well-posed and dissipative boundary conditions are applied.

Since the system is hyperbolic, there exists a matrix of right eigenvectors, $$ {P}$$, and a real diagonal matrix, $$\varLambda $$, such that  for any nonzero space vector , where $$(\hat{x}, \hat{y},\hat{z}) = (\hat{x}_{1}, \hat{x}_{2}, \hat{x}_{3})$$. We also assume that the matrices $$ {A}_{j}$$ are simultaneously symmetrizable and that there exists a piecewise constant matrix $$ {S}$$ such that $$ {A}_{j}^{s}= {S}^{-1} {A}_{j} {S} = \left( {A}_{j}^{s}\right) ^{T}$$.

As a concrete example of the system (1), we pose the linear acoustic wave system where3and where $\rho$ is the density of the medium, *c* is the sound speed, and $\delta _{ij}$ is the Kronecker delta. The state vector can be viewed as representing pressure, *p*, and three velocity components, *u*, *v*, *w*. The coefficient matrices are simultaneously symmetrizable by the matrix

4 $$\begin{aligned} {S} = \left[ \begin{array}{c@{\quad }c@{\quad }c@{\quad }c}c &{} 0 &{} 0 &{} 0 \\ 0 &{} 1/\rho &{} 0 &{} 0 \\ 0 &{} 0 &{} 1/\rho &{} 0 \\ 0 &{} 0 &{} 0 &{} 1/\rho \end{array}\right] . \end{aligned}$$

With jump discontinuities in the material parameters, $\rho$ and *c*, the coefficient matrices and symmetrizer have jump discontinuities.

We contrast the approximation of the system (1) with that of the approximation of systems that can be written in the form5where $$ {E}>0$$ is diagonal and discontinuous at material interfaces while  is continuous. The system (3), for example, can be re-written in the form (5) with symmetric matrices

6 $$\begin{aligned} {E} = \left[ \begin{array}{c@{\quad }c@{\quad }c@{\quad }c}1/\rho c^{2}&{} 0 &{} 0 &{} 0 \\ 0 &{} \rho &{} 0 &{} 0 \\ 0 &{} 0 &{} \rho &{} 0 \\ 0 &{} 0 &{} 0 &{} \rho \end{array}\right] ,\quad {B}_{j} =\left[ \begin{array}{c@{\quad }c@{\quad }c@{\quad }c}0 &{} \delta _{j1} &{} \delta _{j2} &{} \delta _{j3} \\ \delta _{j1} &{} 0 &{} 0 &{} 0 \\ \delta _{j2} &{} 0 &{} 0 &{} 0 \\ \delta _{j3} &{} 0 &{} 0 &{} 0\end{array}\right] , \quad j= 1,2,3 \end{aligned}$$

For equations of the form (5), there is a natural norm,7in which the energy is bounded for homogeneous dissipative physical boundary conditions and nonconservative interface conditions, with that energy satisfying8Stability of DG spectral approximations to equations in the form (5) has been shown specifically, for instance, for Maxwell’s equations [6] and the elastic wave equations [19].

### Remark 2.1

The system (1) cannot in general be rewritten in the form (5). That would require that each $$ {A}_{j}$$ can be written as $$ {A}_{j}= {E}^{-1} {B}_{j}$$ where $$ {E} = {E}^{T}>0$$ and $$ {E}$$ contains all material properties. A counter example is the frozen coefficient compressible Euler equations [1].

**Fig. 2:**
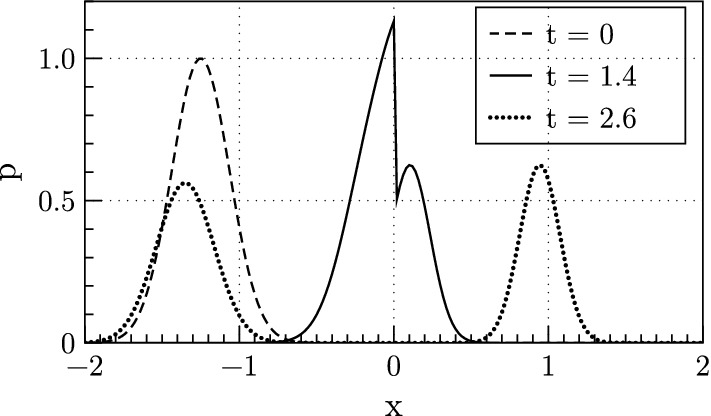
Exact, analytic *p* solution of the one dimensional acoustic wave equation for propagation of a wave across a material interface at $x=0$. The solution is plotted at three times showing the initial incident wave ($t=0$), the interaction with the material discontinuity ($t=1.4$), and the reflected and transmitted waves after the interaction ($t=2.6$)

As noted in [13], systems of the form (1) with discontinuous coefficient matrices do not necessarily have energy bounded by the initial data when measured in the $L_{2}$ norm, and we present an example here to motivate the situation. Figure 2 shows the *p* component of the analytic solution of acoustic wave reflection and transmission at a material boundary placed at $x = 0$ at three times: The initial incident wave, when the wave is interacting with the material discontinuity, and the reflected and transmitted waves after the interaction.

**Fig. 3:**
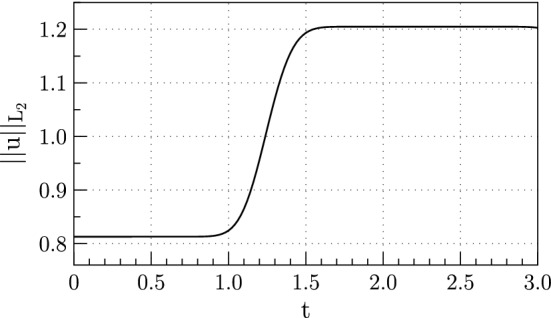
Exact $L_{2}$ energy for the solution of the one dimensional acoustic wave equation for propagation of a wave across a material interface

We plot the energy as a function of time, measured by the $L_{2}$ norm,9in Fig. 3. We see that the $L_{2}$ energy is bounded, and even though the $L_{2}$ energy estimate does not show boundedness directly, energy is bounded in terms of the initial data in a norm that discounts the jump [13]. Note that there is a slight downturn in the energy in Fig. 3 as $t\rightarrow 3$. The energy does decrease to zero after that time as the waves propagate out of the domain.

To establish the stability of the discontinuous Galerkin spectral element approximation of (1), we follow the roadmap presented in [16]. We first establish energy behavior of the PDE system, and then follow an equivalent discrete path to establish an equivalent behavior for the approximation. We begin with the study of the scalar one-dimensional advection problem, since it is easy to follow the steps, and then a symmetric system in one space dimension. Finally we use the symmetric system results to derive the energy bound for the general system in Sect. 2.3.

### Energy Dynamics of the Scalar Problem in One Space Dimension

To motivate (and simplify) the general formulation, we start with the scalar advection equation with two domains as an introduction. Our discussion in this section restates that of [13], but introduces our notation used in succeeding sections.

We derive the energy dynamics of the solution to the scalar advection initial-boundary-value problem in the form (1)

10 $$\begin{aligned} u_{t} + au_{x}= & {} 0 \quad x\in [-1,1]\nonumber \\ u(-1,t)= & {} 0 \nonumber \\ u(x,0)= & {} u_{0}(x), \end{aligned}$$

where

11 $$\begin{aligned} a(x) = \left\{ \begin{gathered} a_{L}> 0\quad x \le 0\\ a_{R}> 0\quad x > 0,\end{gathered} \right. \end{aligned}$$

$a_{L}, a_{R}$ are constants, and $a_{L}\ne a_{R}$. The discussion that follows leads to the same types of conclusions if the wave speeds are both negative. We are interested here in problems where the domains couple and waves propagate from one side to the other. So we do not consider $a_{L}>0,a_{R}<0$, where the domains decouple as energy is dissipated at the interface, or $a_{L}<0,a_{R}>0$ where boundary conditions for both sides are required.

We split the problem into two: Left,

12 $$\begin{aligned} u_{t }+ a_{L}u_{x}= & {} 0 \quad x \le 0\nonumber \\ u(-1,t)= & {} 0,\end{aligned}$$

and Right

13 $$\begin{aligned} u_{t }+ a_{R}u_{x}= & {} 0 \quad x > 0\nonumber \\ u(0^{+},t)= & {} u_{*}(t),\end{aligned}$$

where $u_{*}$ is the upwind specified interface condition chosen so that the Rankine–Hugoniot (or conservation) condition

14 $$\begin{aligned} a_{L}u(0^{-},t) = a_{R}u_{*}(t) \end{aligned}$$

is satisfied. Thus, for the scalar equation, $u_{*}(t) =\frac{a_{L}}{a_{R}} u(0^{-},t)$.

To find the energy equation, we multiply by the solution and integrate over the domains. Define the $L_{2}$ energy norms

15 $$\begin{aligned} \left| \left| u\right| \right| _{L}^{2}= \int _{-1}^{0} u^{2}dx,\quad \left| \left| u\right| \right| _{R}^{2}= \int _{0}^{1} u^{2}dx. \end{aligned}$$

Then

16 $$\begin{aligned} \begin{gathered} \frac{1}{2}\frac{d}{dt}\left| \left| u\right| \right| ^{2}_{L} + \frac{a_{L}}{2}\left. u^{2}\right| _{-1}^{0^{-}} = 0\\ \frac{1}{2}\frac{d}{dt}\left| \left| u\right| \right| ^{2}_{R} + \frac{a_{R}}{2}\left. u^{2}\right| _{0^{+}}^{1} = 0.\end{gathered} \end{aligned}$$

Adding together and re-arranging,

17 $$\begin{aligned} \frac{1}{2}\frac{d}{dt}\left| \left| u\right| \right| ^{2} - \frac{1}{2}a_{L}u^{2}(-1) + \frac{1}{2}\left\{ a_{L}u^{2}(0^{-}) - a_{R}u^{2}(0^{+})\right\} +\frac{1}{2} a_{R}u^{2}(1)=0, \end{aligned}$$

where $\left| \left| \cdot \right| \right| ^{2} = \left| \left| \cdot \right| \right| _{L}^{2} + \left| \left| \cdot \right| \right| _{R}^{2}$. Applying the homogeneous boundary condition on the left,

18 $$\begin{aligned} \frac{1}{2}\frac{d}{dt}\left| \left| u\right| \right| ^{2} + \frac{1}{2}\left\{ a_{L}u^{2}(0^{-}) - a_{R}u^{2}(0^{+})\right\} = -\frac{1}{2} a_{R}u^{2}(1)\le 0. \end{aligned}$$

When we apply the interface condition,

19 $$\begin{aligned} \frac{1}{2}\frac{d}{dt}\left| \left| u\right| \right| ^{2} \le -\frac{1}{2}\left\{ a_{L}u^{2}(0^{-}) - a_{R}u^{2}_{*}\right\} \equiv Q. \end{aligned}$$

The quantity *Q* will be used later in this paper to define stability.

Finally, we substitute the interface value for $u_{*}$,

20 $$\begin{aligned} \frac{1}{2}\frac{d}{dt}\left| \left| u\right| \right| ^{2} \le -\frac{1}{2}\left\{ a_{L}u^{2}(0^{-}) - a_{R}\frac{a_{L}^{2}}{a_{R}^{2}}u^{2}(0^{-})\right\} , \end{aligned}$$

and rearrange so that

21 $$\begin{aligned} \frac{1}{2}\frac{d}{dt}\left| \left| u\right| \right| ^{2} \le -\frac{a_{L}}{2}\left\{ 1 - \frac{a_{L}}{a_{R}}\right\} u^{2}(0^{-}). \end{aligned}$$

Equation (21) shows that the energy is dissipated by the interface only if $a_{R} > a_{L}$. Otherwise the interface generates energy, as illustrated in Fig. 3.

In [13] it was shown that one can construct “discounted norms”, in which the energy is bounded. If the second equation in (16) is multiplied by a constant $\alpha _{c}>0$, then the weighted sum leads to

22 $$\begin{aligned} \frac{1}{2}\frac{d}{dt}\left\{ \left| \left| u\right| \right| ^{2}_{L} + \alpha _{c} \left| \left| u\right| \right| ^{2}_{R}\right\} \le -\frac{a_{L}}{2}\left\{ 1 - \alpha _{c}\frac{a_{L}}{a_{R}}\right\} u^{2}(0^{-}). \end{aligned}$$

Then defining the new norm with the $\alpha _{c}$ discount factor, we have

23 $$\begin{aligned} \frac{d}{dt}\left| \left| u\right| \right| _{\alpha _{c}}^{2}\le 0, \end{aligned}$$

provided that

24 $$\begin{aligned} \alpha _{c}\le \frac{a_{R}}{a_{L}}. \end{aligned}$$

#### Remark 2.2

The weighted norm discounts the effect of the jump, with the result that viewed in the discounted norm, the energy no longer appears to increase. $\square$

#### Remark 2.3

The use of the discounted norm scales to multiple material interfaces and multiple space dimensions by choosing $\alpha _{c}$ to be the minimum over all the ratios of downwind to upwind wave speed ratios. $\square$

#### Remark 2.4

Alternatively, unlike the general case noted in Remark 2.1, the scalar equation (10) can be recast to the form (5) by dividing by the wave speed. Let $\varepsilon = 1/a>0$. Then

25 $$\begin{aligned} \varepsilon u_{t}+ u_{x} = 0. \end{aligned}$$

If the *nonconservative* boundary condition at the interface, $u_{*}=u(0^{+},t) = u(0^{-},t)$, is used, then following the same procedure as (16)–(21),

26 $$\begin{aligned} \frac{1}{2}\frac{d}{dt}\left| \left| u\right| \right| _{\varepsilon }^{2}\le 0, \end{aligned}$$

where the weighted energy norm is given by

27 $$\begin{aligned} \left| \left| u\right| \right| _{\varepsilon }^{2} = \int _{-1}^{0}\varepsilon _{L}u^{2}dx + \int _{0}^{1}\varepsilon _{R}u^{2}dx. \end{aligned}$$

Using the norm (27), but with the conservative interface condition (14), the solution still has a bound like (21), namely

28 $$\begin{aligned} \frac{1}{2}\frac{d}{dt}\left| \left| u\right| \right| _{\varepsilon }^{2}\le -\frac{1}{2}\left\{ 1 - \left( \frac{a_{L}}{a_{R}}\right) ^{2}\right\} u^{2}(0^{-}). \end{aligned}$$

So when the conservative interface condition is used, the weighted energy norm is also bounded only when $a_{L}/a_{R}\le 1$ . $\square$

The discounted norm is equivalent to the $L_2$ norm. In the discounted situation, where $\alpha _c < 1$,

29 $$\begin{aligned} \left| \left| u\right| \right| ^2_{\alpha _c} = \left| \left| u\right| \right| ^2_L + \alpha _c \left| \left| u\right| \right| _R^2\le \left| \left| u\right| \right| ^2_L + \left| \left| u\right| \right| _R^2 = \left| \left| u\right| \right| ^2, \end{aligned}$$

and

30 $$\begin{aligned} \left| \left| u\right| \right| ^2_{\alpha _c} = \alpha _c\left( \frac{1}{\alpha _c}\left| \left| u\right| \right| ^2_L + \left| \left| u\right| \right| _R^2\right) \ge \alpha _c\left( \left| \left| u\right| \right| ^2_L + \left| \left| u\right| \right| _R^2\right) = \alpha _c\left| \left| u\right| \right| ^2. \end{aligned}$$

Therefore,

31 $$\begin{aligned} \sqrt{\alpha _c}\left| \left| u\right| \right| \le \left| \left| u\right| \right| _{\alpha _c}\le \left| \left| u\right| \right| . \end{aligned}$$

Equivalence of the norms means that the $L_2$ norm is actually bounded in terms of the initial data, even though the energy method does not show it directly through (21). From (23) and (31),

32 $$\begin{aligned} \sqrt{\alpha _c}\left| \left| u(T)\right| \right| \le \left| \left| u(T)\right| \right| _{\alpha _c}\le \left| \left| u_0\right| \right| _{\alpha _c}\le \left| \left| u_0\right| \right| . \end{aligned}$$

Thus,

33 $$\begin{aligned} \left| \left| u(T)\right| \right| \le \frac{1}{\sqrt{\alpha _c}}\left| \left| u_0\right| \right| \end{aligned}$$

for the constant coefficient problem.

### Energy Dynamics for Hyperbolic Systems in One Space Dimension

We now increase the complexity and extend the scalar one-dimensional analysis to the general system (1) in one space dimension. We derive the energy equation for the one-dimensional hyperbolic system34where the coefficient matrices are for now assumed to be symmetric. Under this assumption, there is a matrix $$ {P}$$ such that $$ {A} = {P} \varLambda {P}^{-1}$$ satisfying $$ {P}^{-1}= {P}^{T}$$. For the moment, let us assume that $$ {A}$$ has no zero eigenvalues. We also assume that the number of positive and negative eigenvalues does not change across the interface. In other words, there is no eigenvalue that changes sign at the jump. Depending on the sign change, boundary/interface conditions are either lost or gained. More general conditions where the sign of the eigenvalues changes in multi-physics applications are considered in [5]. Finally, we assume that appropriate boundary and initial data are applied.

To find the interface condition at $$x=0$$ for the system (34), we split the system into right and left going waves. The characteristic variables for the system (34) are35where  is associated with the positive eigenvalues of $$ {A}$$ and $$\mathbf{w}^{-}$$ is associated with the negative ones. They are chosen upwind at the interface according to36where here and in the following, the subscripts *R* and *L* correspond to the values at $$x=0^{+}$$ and $$x=0^{-}$$, respectively.

The $\mathbf{w}^{\pm }_{*}$ are computed so that the Rankine–Hugoniot condition37is satisfied at the stationary interface. Let us write

38 $$\begin{aligned} \varLambda = \left[ \begin{array}{c@{\quad }c}\bar{\varLambda }^+ &{} 0 \\ 0 &{} \bar{{\varLambda }}^-\end{array}\right] ,\quad {\varLambda }^{+} = \left[ \begin{array}{c@{\quad }c}\bar{{\varLambda }}^+ &{} 0 \\ 0 &{} 0\end{array}\right] ,\quad {\varLambda }^{-} = \left[ \begin{array}{c@{\quad }c}0 &{} 0\\ 0 &{} \bar{{\varLambda }}^-\end{array}\right] . \end{aligned}$$

Then (37) can be written as39Let us put the unknowns on the left, and the knowns on the right, giving40Equation (40) provides a system of equations for the unknowns.

The matrices on the left of (40) have a special structure since $$ {P}$$ is the matrix of right eigenvectors and $${\varLambda }$$ is a diagonal matrix. Let *n* be the number of positive eigenvalues out of a total of *m*. Then define41and42 where  is the eigenvector associated with the eigenvalue $$\lambda _{j}$$ and the eigenvalues are ordered in decreasing order, largest to smallest with $$\lambda _{j}>0$$ for $$j\le n$$. Then we can write (40) as43Given the structure of the $$ {M}^{\pm }$$ matrices, the equations can be combined to produce a single system for the unknowns44where

45 $$\begin{aligned} {M}_{LR}\equiv {M}^{-}_{L} - {M}^{+}_{R},\quad {M}_{RL}\equiv {M}^{-}_{R} - {M}^{+}_{L}. \end{aligned}$$

Existence and uniqueness of the inflow characteristic vectors $$\mathbf{w}_{*}^{\pm }$$ therefore depends on the existence of the inverse of the matrix $$ {M}_{LR}$$. That matrix is comprised of eigenvectors of the coefficient matrix evaluated on the left and eigenvectors evaluated on the right. On the one hand, if the eigenvectors of the coefficient matrix do not change across the material discontinuity, then, since the eigenvectors are independent, $$ {M}_{LR}^{-1} {M}_{LR}$$ is diagonal. As an example, the eigenvectors of the acoustic wave system (3) are constant, being independent of the material properties on either side. On the other hand, if the eigenvectors change across the interface and the matrix $$ {M}_{LR}^{-1} {M}_{RL}$$ is not diagonal, then the problem is ill-posed [5]. We therefore require that the eigenvectors be preserved across the jumps so that $$ {M}_{LR}^{-1}$$ exists, $$ {M} \equiv {M}_{LR}^{-1} {M}_{LR}$$ is diagonal, and46

#### Remark 2.5

The Rankine–Hugoniot (conservation) condition (37) limits the form of the interface condition significantly. If only boundedness is desired, more general coupling conditions are allowed [5]. $\square$

#### Remark 2.6

One can see that the assumption that there are no zero eigenvalues is not a restriction. If there are zero eigenvalues, then the associated characteristic variables $\mathbf{w}^{0}$ are multiplied by the zero matrix and have no contribution to the system. Therefore those quantities can be eliminated, leaving (43) and what followed. The $\mathbf{w}^{0}$ vector is determined by the initial data. $\square$

Going back to the original equations, (34), we compute the energy equation by multiplying by the state and integrating over the domain, giving47where, now, $\left| \left| \mathbf{u}\right| \right| ^{2} = \left\langle \mathbf{u},\mathbf{u}\right\rangle$ and ${\,{\text {PBT}}}$ represents the terms coming from the physical boundary conditions on the left and right. Since we are only interested here in the interface conditions, we will assume that the physical boundary conditions are well posed so that ${\,{\text {PBT}}}\ge 0$. In that case,48where49is the interface contribution to the energy.

Following the steps in the scalar analysis, we now apply the interface boundary conditions on *Q*. We decompose the system into characteristic variables. Then we use the fact that $$ {A}$$ is symmetric, making $$ {P}^{-1}= {P}^{T}$$. With this decomposition,50taking into account the upwinding of the characteristic variables, (36).

We now gather the right-going and left-going wave contributions (c.f. (19)),51and then use the fact that $\bar{{\varLambda }}^{-}<0$, to get the final form of the interface contribution, which we write in terms of its characteristic components,52Equation (52) is the system version of the scalar interface condition seen in (19).

As in the scalar problem, one can construct a discounted norm $$\left| \left| \cdot \right| \right| _{B}$$ for which the associated interface term $$Q_{B}$$ is non-positive and the discounted norm is bounded when the coupling matrix, $$ {M}$$, exists and is diagonal. For instance, one simple choice is to let

53 $$\begin{aligned} {B} = {P}\left[ \begin{array}{c@{\quad }c@{\quad }c@{\quad }c@{\quad }c@{\quad }c}\mu ^+ &{} &{} &{} &{} &{} \\ &{} \ddots &{} &{} &{} &{} \\ &{} &{} \mu ^+ &{} &{} &{} \\ &{} &{} &{} \mu ^- &{} &{} \\ &{} &{} &{} &{} \ddots &{} \\ &{} &{} &{} &{} &{} \mu ^-\end{array}\right] {P}^{-1}, \end{aligned}$$

where the entries with $$\mu ^{\pm }>0$$ are counted according to the number of positive and negative eigenvalues of $$ {A}$$. Then multiplying the system on $$x>0$$ by $$ {B}$$ from the left, defining the norm , and following the same steps leading to (52), the interface contribution to the energy is54One then only needs to find $$\mu ^{+}$$ small enough and $$\mu ^{-}$$ large enough to ensure that $$Q_{B}\le 0$$, in which case the new energy  is bounded in terms of the initial data. Since the coupling matrix $$ {M}$$ is diagonal, let us split it as

55 $$\begin{aligned} {M} = \left( \begin{array}{c@{\quad }c}\bar{M}^+ &{} 0 \\ 0 &{} \bar{M}^-\end{array}\right) \end{aligned}$$

so that  and . Then $Q_{B}\le 0$ if $\mu ^{\pm }$ are chosen so that

56 $$\begin{aligned} \begin{aligned} \bar{{\varLambda }}_{L}^{+} - \mu ^{+}\bar{M}^{+,T}{\varLambda }_{R}^{+}\bar{M}^{+}&>0,\\ \mu ^{-}\left| \bar{{\varLambda }}_{R}^{-}\right| - \bar{M}^{-,T}\left| \bar{{\varLambda }}_{L}^{-}\right| \bar{M}^{-}&>0. \end{aligned} \end{aligned}$$

### Extension to Non-symmetric Equations and an Arbitrary Domain

The results of the previous two sections extend to general geometries and non-symmetric coefficient matrices. In preparation for the generalization of (52), we note that within each subdomain the coefficient matrices are constant, and therefore we can re-write (1) in a split form as57We also define the inner product and norm over a subdomain $$D = {\varOmega }_{L}$$ or $$ {\varOmega }_{R}$$ as58so that59To form the energy, we take the inner product of (57) with the vector , giving60Let us define  to be the symmetric system state. Then since $$ {S}$$ is constant within the subdomains and $$ {A}^{s}= {S}^{-1} {A} {S} $$,61We then apply multidimensional integration by parts and symmetry to the divergence term62where  is the outward normal at the boundary of *D*, and note that the volume term cancels the third term in (61), leaving only the boundary integral,63Then over the domain $${\varOmega }$$,64where *L*/*R* represent the states on either side of the interface with respect to the normal .

We can now get a bound for the multidimensional system similar to (48). The integrand in the interface integral is identical to that in (47), with  and $\mathbf{u} \leftarrow \mathbf{u}^{s}$. Therefore, if the boundary conditions along ${\varGamma }_{b}$ are properly posed and dissipative,

65 $$\begin{aligned} \frac{1}{2}\frac{d}{dt}\left| \left| \mathbf{u}^{s}\right| \right| _{{\varOmega }}^{2}\le \int _{{\varGamma }}Q{\,{\text {dS}}}. \end{aligned}$$

Therefore, *Q* is still given by (52), but now formulated in the new symmetrized variables. Note that the norm defined by $\left| \left| \mathbf{u}^{s}\right| \right| ^{2} = \left\langle \left( {S}^{-1}\right) ^{T} {S}^{-1}\mathbf{u},\mathbf{u}\right\rangle$ is equivalent to the norm $\left| \left| \mathbf{u}\right| \right|$ since $\left( {S}^{-1}\right) ^{T} {S}^{-1}>0$.

### Stability

In summary, for hyperbolic systems of the form (1), with discontinuities in the coefficient matrices and homogeneous, dissipative boundary conditions, the $L_{2}$ norm of the solution obeys (65). The integrand of the interface contribution, *Q*, is of the form (52), where the characteristic variables are evaluated from the upwind side and satisfy the Rankine–Hugoniot condition. It is not necessarily non-negative, depending on the relative wave speeds from either side of the interface, so the $L_{2}$ norm of the solution is not bounded in general by the initial data. An example of such behavior was shown in Fig. 3.

Although the $L_{2}$ norm (or, for that matter, weighted norms, see Remark 2.4) is not always bounded in terms of the initial data, there exists an energy in a discounted norm that is bounded in the usual way provided that the coupling matrix between the upwind and downwind states is diagonal.

Thus, we have two views of stability at our disposal, which we will call direct and inferred:

- **Direct Stability** When the $L_{2}$ norm is bounded, we directly have $L_{2}$ stability. This is seen in scalar problems if $a_{L} / a_{R}\le 1$ in (21). For the system, the equivalent is when $\bar{{\varLambda }}_{L}^{+} - \bar{M}^{+,T}{\varLambda }_{R}^{+}\bar{M}^{+}>0$ and $\left| \bar{{\varLambda }}_{R}^{-}\right| - \bar{M}^{-,T}\left| \bar{{\varLambda }}_{L}^{-}\right| \bar{M}^{-}>0$, as seen through (56) setting $\mu ^{\pm } = 1$.
- **Inferred Stability** Nonetheless, even if the $L_{2}$ norm is not directly bounded, we have seen that one can construct a discounted norm in which it is, e.g. (23) for scalar problems and for systems when (56) is satisfied. Stability in some discounted norm is therefore inferred, or implicit, if *Q* is given by (52).

In general geometries it may not be easy to find the discounted norm in which the solution is bounded. Finding the precise coefficients requires satisfying conditions like (56). When multiple subdomains exist in multiple space dimensions, *T*-type intersections between materials are possible. The discount factors must then take into account all subdomain boundaries and be adjusted globally so that at each interface (56) is still satisfied. For these reasons, it is easier to monitor the behavior (65) of the simpler $L_{2}$ norm as a surrogate to infer well-posedness of the system. Further insights into how choosing the norm affects how the energy is bounded or not can be found in [14].

Stability of a numerical approximation of a system follows that of the PDE, and so we state the stability condition for the approximation as:

#### Definition 2.1

A scheme approximating the discontinuous coefficient problem (1) is said to have inferred stability if the discrete approximation of the standard $L_{2}$ norm is bounded as in (65) and the approximation to the integrand, $Q_{N} \approx Q$, satisfieswhere $\mathbf{W}$ is the approximation of $\mathbf{w}$.

## The Discontinuous Galerkin Spectral Element Discretization

In this section, we briefly summarize the important discretization steps. For a detailed description and derivation of the scheme, we refer to e.g. [4, 10, 21].

The first step is to divide the computational domain into a mesh of non-overlapping, possibly curved, hexahedral (quadrilateral in 2D) elements, $\{e^l\}_{l=1}^K$. Each hexahedron is mapped from physical space to a reference space cube $E = [-1,1]^3$ with . To retain spectral accuracy and exponential convergence in the presence of jump discontinuities, we require element faces to be aligned with the material interface, ${\varGamma }$, so that polynomial approximation is not made across the discontinuity.

From the mapping, we can compute the metric terms66Note that we need to carefully evaluate the metric terms to get a discretely divergence-free contravariant basis , which is necessary to guarantee free-stream preservation of the discretization [7] and stability of the volume terms [4, 12].

The second step of the discetization process is to transform the problem (57) from physical to reference space. In reference space, (57) becomes67where we collect the metric terms in the block matrix

68 $$\begin{aligned} \mathfrak M = \begin{pmatrix} Ja_1^1\, {I} &{} \quad Ja_1^2\, {I} &{} \quad Ja_1^3\, {I}\\ Ja_2^1\, {I} &{}\quad Ja_2^2\, {I} &{} \quad Ja_2^3\, {I}\\ Ja_3^1\, {I} &{} \quad Ja_3^2\, {I} &{} \quad Ja_3^3\, {I}\\ \end{pmatrix}, \end{aligned}$$

with the identity matrix, $$ {I}$$, having the size as the state vector $$\mathbf{u}$$.

The third step is the variational Galerkin formulation. We first approximate the solution with an interpolatory polynomial of degree *N*, and denote polynomial approximations with capital letters ${u\approx U = \mathbb {I}^N(u)}$, where $\mathbb {I}^N$ denotes the interpolation operator. In the spectral collocation framework, one typically uses a nodal basis for the interpolation. Furthermore, for hexahedral/quadrilateral elements, we use a tensor-product of one-dimensional nodal Lagrange basis functions spanned on the Legendre–Gauss–Lobatto nodes. The same polynomial approximation is used for all quantities, e.g. for the contravariant flux function .

To get the variational formulation, we multiply the transformed PDE (67) by polynomial test functions $\varvec{\varphi }$, which are linear combinations of the nodal basis functions. Then we integrate over the reference element *E* and use integration-by-parts to arrive at69where $\hat{n}$ is the reference space outward pointing normal vector to the face $\partial E$.

Finally, we replace the integration in (69) by quadrature and cubature rules, collocated with the Legendre–Gauss–Lobatto interpolation. Note, that the Legendre–Gauss–Lobatto nodes include the boundary nodes and hence surface and volume integration nodes partially coincide with the interpolation ansatz and $\mathbb {I}^N(\cdot )$ can be dropped. Furthermore, we introduce the yet to be defined numerical flux function , which depends on the two states $\mathbf{U}^{L,R}$ at the interface and approximates the normal flux through the interface. Note that we assume the coefficients ${ {A}}$ are mostly constant, but when they jump, the mesh is aligned so that an element interface is at the jump. Hence, the numerical flux function at the coefficient jump interface depends not only on the solutions left and right, but also on the coefficients left and right: $\mathbf{F}_n^*=\mathbf{F}_n^*(\mathbf{U}^{L,R};{ {A}}^{L,R})$.

Applying quadrature, we get the formal statement of the DGSEM,70where $\left\langle \cdot ,\cdot \right\rangle _{N}$ and $\int \limits _{\partial E,N}$ represent the volume and surface quadratures, see [11]. The right hand side of (70) is written in terms of the normal covariant fluxes and is equivalent to that written in terms of the contravariant ones [21]. The resulting high-order semi-discretization is integrated with a proper high-order accurate explicit Runge–Kutta time integrator, which is stable under the typical CFL-type time step restriction.

## Stability of the Discontinuous Galerkin Approximation

We establish the stability bound from the weak form of the equation, (70). We then follow the path taken in Sect. 2 for the continuous problem to examine the discontinuous interface term: We examine the scalar problem for insights, then the symmetric one-dimensional system, and finally the general problem for the DGSEM approximation.

### Discrete Stability Estimate

For a detailed derivation of the discrete stability estimate, which parallels the continuous analysis, we refer to [4, 21]. Here, we will only sketch some important intermediate steps. To get the stability estimate, we replace the test function $$\varvec{\varphi }$$ with the approximate solution polynomial and the symmetrizer matrices, writing $$\varvec{\varphi }= \left( {S}^{-1}\right) ^{T} {S}^{-1}\mathbf{U} = \left( {S}^{-1}\right) ^{T}\mathbf{U}^s $$ to get71where we define the symmetrized discrete flux $$\mathbf{F}^s_n$$ that uses the symmetric coefficient matrices . Using the fact that the symmetrizer matrix $$ {S}$$ commutes with the metric block matrix $$\mathfrak M$$ (see e.g. [4, 21]) we see that the volume terms cancel, leaving only surface terms,

72 $$\begin{aligned} \begin{aligned} \left\langle J\,\mathbf{U}^s_t,\mathbf{U}^s\right\rangle _N =&- \int \limits _{\partial E,N} (\mathbf{U}^s)^T\left\{ \mathbf{F}_n^{s,*} - \frac{1}{2}\mathbf{F}^s_n\right\} \,{\,{\text {dS}}}. \end{aligned} \end{aligned}$$

When we sum over all elements, inner surface terms appear twice (with different normal vectors), whereas element surfaces that are at the physical domain boundary appear only once and are denoted as physical boundary terms (${\,{\text {PBT}}}$). The interior element surface contributions split into two parts: Surfaces that fall on the material interface ${\varGamma }$, and those across which the coefficient matrices are the same, which we call smooth interface boundary terms, ${\text {SIBT}}$. The sum over all elements can then be written as73written in terms of the jump operator, . Assuming that the discrete physical boundary terms are dissipative, the discrete $L_{2}$ norm satisfies74which mimics the continuous stability (65) if ${\text {SIBT}}\le 0$.

We thus need a proper numerical flux function $\mathbf{F}_n^{s,*}$ to control discrete stability, i.e. to guarantee that the integrand satisfies75pointwise at each node on element faces along the discretization of ${\varGamma }$, and ${\text {SIBT}}\le 0$.

The dissipativity of the ${\text {SIBT}}$ for the upwind numerical flux has been shown elsewhere, e.g. [8, 21]. Therefore, in the following we will assume ${\text {SIBT}}\le 0$ and concern ourselves only with the discontinuous interface terms.

### Stability for the Scalar Problem

In the DG approximation, the Rankine–Hugoniot condition and the inflow boundary condition are enforced weakly with the upwind numerical flux

76 $$\begin{aligned} F^{*}(U_{L},U_{R}; a_{L},a_{R}) = a_{L} U_{L}, \end{aligned}$$

If summation by parts is applied again to the second term in (70), one gets the strong form of the approximation, in which the integrand of the boundary term is [21]

77 $$\begin{aligned} F^{*}-F = a_{L}U_{L}- a_{R}U_{R}. \end{aligned}$$

As the solution converges, this difference goes to zero, and the Rankine–Hugoniot condition is satisfied. Furthermore,

78 $$\begin{aligned} F^{*}-F = a_{L}U_{L}- a_{R}U_{R} = a_{R}\left( \frac{a_{L}}{a_{R}}U_{L} -U_{R} \right) , \end{aligned}$$

so that when the approximation converges, the analytical inflow boundary condition, $U_{R} = \frac{a_{L}}{a_{R}}U_{L}$ is approached, as required, c.f. (14).

With (76), the interface contribution for the scalar problem is

79 $$\begin{aligned} \begin{aligned} Q_{N}&= (U_{R}-U_{L})a_{L} U_{L} - \frac{1}{2}(a_{R}U^{2}_{R}- a_{L}U_{L}^{2})\\ {}&= U_{R}a_{L}U_{L} - a_{L}U_{L}^{2}-\frac{1}{2}a_{R}U^{2}_{R} + \frac{1}{2}a_{L}U_{L}^{2}\\ {}&= -\frac{1}{2}\left( a_{L}U_{L}^{2} - 2 U_{R}a_{L}U_{L} + a_{R}U^{2}_{R} \right) . \end{aligned} \end{aligned}$$

Factoring the quadratic,

80 $$\begin{aligned} \begin{aligned} Q_{N}&= -\frac{1}{2}\left( a_{L}U_{L}^{2} - 2 U_{R}a_{L}U_{L} + a_{R}U^{2}_{R} \right) \\ {}&= -\frac{1}{2}a_{L}U_{L}^{2}\left( 1 - 2\frac{U_{R}}{U_{L}} + \frac{a_{R}}{a_{L}}\left( \frac{U_{R}}{U_{L}}\right) ^{2}\right) \\ {}&= -\frac{1}{2}a_{L}U_{L}^{2}\tilde{Q}\left( \frac{U_{R}}{U_{L}};\frac{a_{L}}{a_{R}} \right) . \end{aligned} \end{aligned}$$

The quadratic $\tilde{Q}(\eta ;\frac{a_{L}}{a_{R}})$ is concave up and has a minimum when $\eta ^{*} = a_{L}/a_{R}$, since

81 $$\begin{aligned} \tilde{Q}'=-2 + 2\frac{a_{R}}{a_{L}} \eta ,\quad \tilde{Q}'' = 2\frac{a_{R}}{a_{L}} >0. \end{aligned}$$

When $\eta ^{*} = a_{L}/a_{R}$, the Rankine–Hugoniot condition is satisfied by the states on either side. The value of that minimum is $\tilde{Q}(\eta ^{*};\frac{a_{L}}{a_{R}}) = 1 - \frac{a_{L}}{a_{R}}$.

It then follows that the contribution to the energy in the numerical approximation matches that of the PDE, (21), plus a dissipation term dependent on how much the Rankine–Hugoniot condition is not satisfied by the approximate solution. If we define $\beta = a_{L}/a_{R}$, and note that the minimum value of $\tilde{Q}$ is $1-\beta$, we can separate out that term giving

82 $$\begin{aligned} \begin{aligned} \tilde{Q}(\eta ;\beta ) = 1-2\eta +\frac{1}{\beta }\eta ^{2}&= (1-\beta ) + (1-2\eta +\frac{1}{\beta }\eta ^{2}) - (1-\beta ) \\ {}&= (1-\beta ) + \frac{1}{\beta }(\eta -\beta )^{2}. \end{aligned} \end{aligned}$$

Re-writing the interface contribution in the final form of (82) will be a key step in showing inferred stability of the approximation for the more complex case of a system of equations.

When we substitute for $\eta$ and $\beta$,

83 $$\begin{aligned} \begin{aligned} Q_{N}&= -\frac{1}{2}a_{L}U_{L}^{2}\left( 1 - \frac{a_{L}}{a_{R}}\right) - \frac{a_{R}U_{L}^{2}}{2}\left( \frac{U_{R}}{U_{L}} - \frac{a_{L}}{a_{R}}\right) ^{2} \\ {}&= -\frac{1}{2}a_{L}\left( 1 - \frac{a_{L}}{a_{R}}\right) U_{L}^{2} -\frac{1}{2a_{R}}\left( a_{R}U_{R}- a_{L}U_{L}\right) ^{2}. \end{aligned} \end{aligned}$$

Let us compare: In the continuous case, we have (21), with

84 $$\begin{aligned} Q\left( u(0^{-}),u(0^{+})\right) = -\frac{a_{L}}{2}\left\{ 1 - \frac{a_{L}}{a_{R}}\right\} u^{2}(0^{-}), \end{aligned}$$

whereas discretely,

85 $$\begin{aligned} Q_{N}=Q\left( U_{L},U_{R}\right) -\frac{1}{2a_{R}}\left( a_{R}U_{R}- a_{L}U_{L}\right) ^{2}\le Q\left( U_{L},U_{R}\right) . \end{aligned}$$

Thus, according to the definition of stability, Definition 2.1, the DGSEM approximation of the scalar problem with the upwind numerical flux has inferred stability.

#### Remark 4.1

The comparison between (84) and (85) shows explicitly what is interpreted as stability. The first term in (85) can be positive or negative depending on $a_{L}/a_{R}$, but matches that of the PDE, (84). The approximation is therefore directly stable if $a_{L}/a_{R}\le 1$, just like the PDE. The second term is always non-positive and represents dissipation of the energy by the approximation. $\square$

#### Remark 4.2

For the scalar problem it is straightforward, as for the continuous problem, to show energy boundedness in a discounted norm by scaling the downwind domain contributions before summing over the elements. When the global sum (in this case, over two elements) is formed, the parenthetical term in the second line of (80) becomes

86 $$\begin{aligned} \tilde{Q}_{N,\alpha _{c}}=\left( 1 - 2\alpha _{c}\frac{U_{R}}{U_{L}} + \alpha _{c}\frac{a_{R}}{a_{L}}\left( \frac{U_{R}}{U_{L}}\right) ^{2}\right) . \end{aligned}$$

Like the original quantity, $\tilde{Q}_{N}$ in (80), $\tilde{Q}_{N,\alpha _{c}}$ is concave up, with minimum at the same point, $\eta ^{*}$, with minimum value

87 $$\begin{aligned} \tilde{Q}_{N,\alpha _{c}}(\eta ^{*};\frac{a_{L}}{a_{R}}) = 1 - \alpha _{c}\frac{a_{L}}{a_{R}}, \end{aligned}$$

so

88 $$\begin{aligned} \tilde{Q}_{N,\alpha _{c}}(\eta ;\frac{a_{L}}{a_{R}}) \ge 1 - \alpha _{c}\frac{a_{L}}{a_{R}}. \end{aligned}$$

Since one can always show bounded energy in the new discounted norm by choosing $\alpha _{c}$ to match the analytical value for any (positive) wavespeeds, the condition (85) infers stability. The amount of numerical dissipation in that norm depends on the particular choice of $\alpha _{c}$, however. $\square$

### Stability for the One-Dimensional Symmetric System

We now parallel Sect. 2.2 and extend the analysis to a symmetric PDE system in one space dimension. For the system, the DG approximation has the interface contribution89The upwind numerical flux is now90with the equalities between the left and right representations arising by virtue of the Rankine–Hugoniot condition. The key observation is that91But  and for the symmetric system $$ {P} ^{T} {P} = {I}$$, so92Now,93and94Therefore,95Looking at the second jump term in (89),96so97Therefore, forming $$Q_{N}$$ and gathering right and left going wave components,98Terms cancel, leaving99Following (82), we now add and subtract terms to match the PDE form, which is100to write101Now, let102Then103To show that the approximation is stable according to Definition 2.1, then, we just need to show that $$R^{\pm }\ge 0$$, since the other terms match those of the PDE. To do so, let . Then104Similarly,105Thus, the interface contribution matches that of the PDE plus an additional dissipation and has inferred stability, satisfying Definition 2.1 with106

### Stability of the General Problem

As in the continuous problem, we use the analysis of the one-dimensional problem to imply stability of the multidimensional one. As before, replace $\mathbf{U} \leftarrow \mathbf{U}^{s}$ and . Then $Q_{N}$ is given by (106), with the eigenvalues (and eigenvectors to construct the characteristic variables) coming from . Therefore the approximation to the general multidimensional problem is stable according to Definition 2.1.

#### Remark 4.3

The key features of the stability analysis are the use of summation by parts, and a stable implementation of the boundary terms. As such, the analysis extends to other methods that have the summation by parts property and allow discontinuities at subdomain interfaces, such as summation by parts finite difference techniques, e.g. as used in [15, 17, 18].

## Example

As an example, we consider the scattering of a plane wave off a plane material interface, approximating the system of equations (1) with the state vector and coefficient matrices (3) reduced to two space dimensions. The problem has exact incident, transmitted and reflected plane wave solutions of the form107where $\psi$ is a given wavefunction, *a* is the amplitude,  is the wavevector, $\omega$ is the frequency. For the incident wavevector108the reflected and transmitted wavevectors are109with amplitudes110where111For the wavefunction, we choose the Gaussian

112 $$\begin{aligned} \psi (s) = e^{-s^{2}/(\omega \sigma )^{2}}, \end{aligned}$$

with $\sigma ^{2} = -(MT)^{2}/(4\ln (10^{-4}))$, $M = 4$ and period $T = 2\pi /\omega$.

We compute the problem on the square domain $[-5,5]^{2}$ with 400 square elements and the material interface at $x=0$. The solution parameters are provided in Table 1.

**Table 1:** Parameters for the plane wave reflection problem

Parameter	*M*	$\omega$	$k^i_x$	$k^i_y$	$\rho _L$	$\rho _R$	$c_L$	$c_R$	$t_{0}$
Value	4	$4\pi$	0.5	$\sqrt{3/2}$	1	0.4	1	0.7	3

**Fig. 4:**
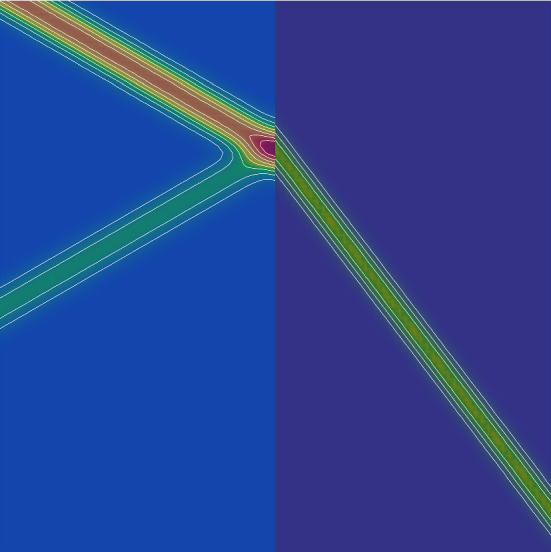
Computed *p* contours at time $t=5$ for plane wave scattering from a material interface along the vertical center of the domain

**Fig. 5:**
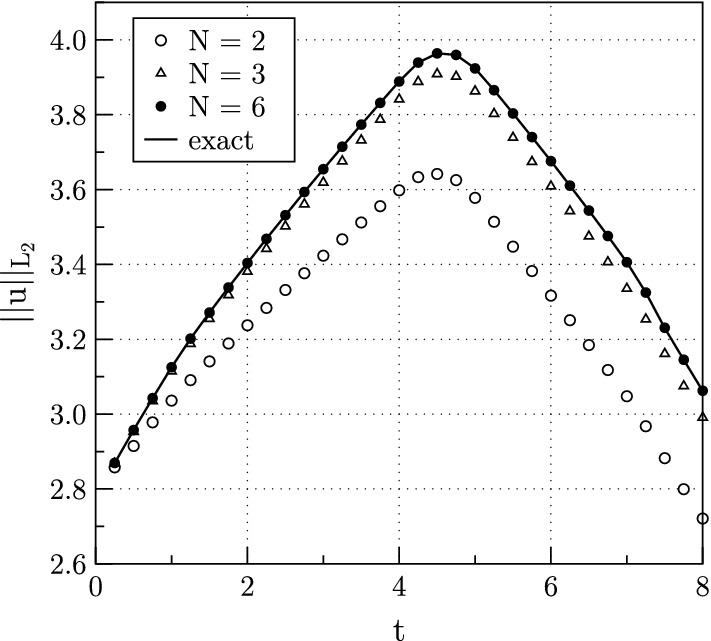
$L_{2}$ energy as a function of time for scattering at a material interface

The results are shown in Figs. 4 and 5. Figure 4 shows the contours of the *p* component of the solution at time $t=5.0$, which is near the time of the maximum $L_{2}$ energy, computed with sixth order polynomials. Clearly seen is the jump discontinuity at the interface. The $L_{2}$ energy is plotted as a function of time in Fig. 5, for polynomial degrees $N= 2, 3$ and 6. Although the $L_{2}$ energy initially grows, it reaches a maximum around time $t=4.5$. Figure 5 shows that the computed energy converges from below to the exact as the polynomial order is increased. In fact, it converges exponentially with polynomial degree, as expected [2] for a spectral element method. Also, as expected due to the additional dissipation at physical, smooth and discontinuous interfaces, the computed energies fall below the exact curve and are worst for low order approximations.

## Conclusions

We have shown that the interface treatment of the discontinuous Galerkin spectral element method with the upwind numerical flux is stable for hyperbolic systems with discontinuous coefficient matrices when the eigenvectors are preserved across the interface. Examples include systems like Maxwell’s equations, or acoustic and elastic wave equations. The new feature of our approach was to show that the discrete $L_{2}$ norm of the approximate solution grows no faster than the same norm of the continuous solution. By matching the $L_{2}$ norm, we avoid having to find the precise conditions for a discounted norm in which the energy is bounded in terms of the initial data (for homogenous and dissipative boundary conditions). The numerical flux only weakly enforces the inflow boundary condition and the Rankine–Hugoniot condition. Viewing stability in terms of the $L_{2}$ norm shows that the dissipation introduced by the upwind numerical flux depends on the amount by which the approximate solution fails to satisfy the Rankine–Hugoniot condition.

## Data Availability

All relevant data generated or analysed during this study are included in this published article.

## Declaration

### Code Availability

The code used to generate the results in this work is available upon request from David A. Kopriva (kopriva@math.fsu.edu).
